# Influence of Oxidation of Copper on Shear Bond Strength to an Acrylic Resin Using an Organic Sulfur Compound

**DOI:** 10.3390/ma13092092

**Published:** 2020-05-01

**Authors:** Haruto Hiraba, Hiroyasu Koizumi, Akihisa Kodaira, Hiroshi Nogawa, Takayuki Yoneyama, Hideo Matsumura

**Affiliations:** 1 Department of Fixed Prosthodontics, Nihon University School of Dentistry, 1-8-13, Kanda-Surugadai, Chiyoda-ku, Tokyo 101-8310, Japan; hiraba.haruto@nihon-u.ac.jp (H.H.); kodaira.akihisa@nihon-u.ac.jp (A.K.); nogawa.hiroshi@nihon-u.ac.jp (H.N.); matsumura.hideo@nihon-u.ac.jp (H.M.); 2 Division of Advanced Dental Treatment, Dental Research Center, Nihon University School of Dentistry, 1-8-13, Kanda-Surugadai, Chiyoda-ku, Tokyo 101-8310, Japan; 3 Department of Dental Materials, Nihon University School of Dentistry, 1-8-13, Kanda-Surugadai, Chiyoda-ku, Tokyo 101-8310, Japan; yoneyama.takayuki@nihon-u.ac.jp; 4 Division of Biomaterials Science, Dental Research Center, Nihon University School of Dentistry, 1-8-13 Kanda-Surugadai, Chiyoda-ku, Tokyo 101-8310, Japan; 5 Applied Oral Sciences & Community Dental Care, Faculty of Dentistry, The University of Hong Kong, Hong Kong SAR 999077, China

**Keywords:** acrylic resin, bond strength, dental metal, oxide film, organic sulfur compound

## Abstract

The aim of this study was to clarify the influence of the copper surfaces changed from Cu or Cu_2_O to CuO on the bonding strength of resin with organic sulfur compounds. The disk-shaped specimens (*n* = 44) of copper were wet-ground. Half of the specimens were heated at 400 °C for 4 min in an electric furnace (HT: heated). Half of the specimens were not heated (UH: unheated). The specimens were further divided into two groups. Each group was primed by 6-methacryloyloxyhexyl 2-thiouracil-5-carboxylate (MTU-6) or unprimed (*n* = 11). A statistical analysis of the results of shear bond strength testing was performed, and the failure mode of the bonded areas was classified with an optical microscope. Two types of specimen surface (UH or HT) were analyzed chemically using X-ray photoelectron spectroscopy (XPS). When primed with MTU-6, unheated Cu (28.3 MPa) showed greater bond strength than heated (19.1 MPa). When unprimed, heated Cu (4.1 MPa) showed greater bond strength than unheated (2.3 MPa). The results of the debonded surfaces observation showed that only the UH-MTU-6 group demonstrated a combination of adhesive and cohesive failures in all specimens. The XPS results showed that the surface of copper changed from Cu or Cu_2_O to CuO when HT. These results confirmed that it is necessary to take care of the copper oxide contained in noble metal alloys when using organic sulfur compounds for adhesion.

## 1. Introduction

Gold alloys with excellent workability and strength are widely and routinely used in dental practice. The strength of gold alloys, in particular type III and IV, is improved by copper. Copper has the property of forming an oxide film on the surface by gradually reacting with atmospheric oxygen. The surface of copper changes from Cu to Cu_2_O, and further heating forms CuO [1]. The oxidation is generally controlled by alloying, whereas copper included in alloys is oxidized by heating [2,3]. Equally important, is the fact that copper is a metal that is strongly associated with adhesion.

Adhesive functional monomers, e.g., carboxylic acids, phosphates, or organic sulfur compounds, are used for greater bonding to dental metals. Carboxylic acids and phosphates of acidic functional monomers act on the oxide film formed on non-noble metals, and therefore produce no effect on bonding to noble metals that do not easily form the oxide film [4,5,6,7]. Tanaka et al. focused on the oxidation of copper in order to solve this problem, and reported that the effects of carboxylic acids included in acrylic resin were improved by oxidation treatment for a noble metal alloy [8,9]. Organic sulfur compounds acting directly on noble metals originated from the study of the corrosion resistance of a copper plate in 1983 [10]. Organic sulfur compounds, especially 6-methacryloyloxyhexyl 2-thiouracil-5-carboxylate (MTU-6), have been demonstrated to show a stable high bonding strength for noble metal alloys [5,6,9,11]. The bonding mechanism involves organic sulfur compounds such as MTU-6 being strongly adsorbed onto noble metals by the tautomerization structure of the thione–thiol type and copolymerized with an acrylic resin [4,7]. MTU-6 adsorbed on the surface of the noble metal alloy, despite being rinsed with acetone, was detected by X-ray photoelectron spectroscopy (XPS) [6]. Yamashita et al. reported that MTU-6 was superior to other organic sulfur compounds in the bonding performance of copper, simultaneously suggesting that corrosion of copper is concerned with bond durability [5].

Airborne-particle abrasion is also recognized as an easy surface modification method. An airborne-particle abrasion with alumina was recently shown to not only bring about surface cleaning and mechanical roughening, but may also oxidize copper included in the noble metal alloy [9,12]. Those studies suggested that copper oxide is effective when used with primers containing both an acidic functional monomer and an organic sulfur compound. However, the effects of the oxidation of copper on the bonding with organic sulfur compounds are not clear. The effects of the oxidation of copper might be concealed by other noble metals, such as gold and silver, including in alloys, based on the results of previous studies with noble metal alloys [9,12]. The null hypothesis was that the CuO film had no adverse effects on MTU-6 in adhesion of an acrylic resin and copper. Therefore, this study was conducted to clarify the influence of the copper surfaces changed from Cu or Cu_2_O to CuO on the organic sulfur compound (MTU-6) and its effectiveness. 

## 2. Materials and Methods

### 2.1. Materials

Copper metal (Nilaco Corp., Tokyo, Japan) was used as an adherend material. Metaltite (Tokuyama Dental Corp., Tokyo, Japan) containing 6-methacryloyloxyhexyl 2-thiouracil-5-carboxylate (MTU-6) in ethanol was used for priming. Tri-*n*-butylborane (TBB; Super-Bond C&B Catalyst V, Sun Medical Co., Ltd., Moriyama, Japan) was used as the polymerization initiator of a methyl methacrylate (MMA)-based acrylic resin (MMA-TBB resin). These materials are listed in Table 1.

### 2.2. Preparation of Specimens

**Figure 1:**
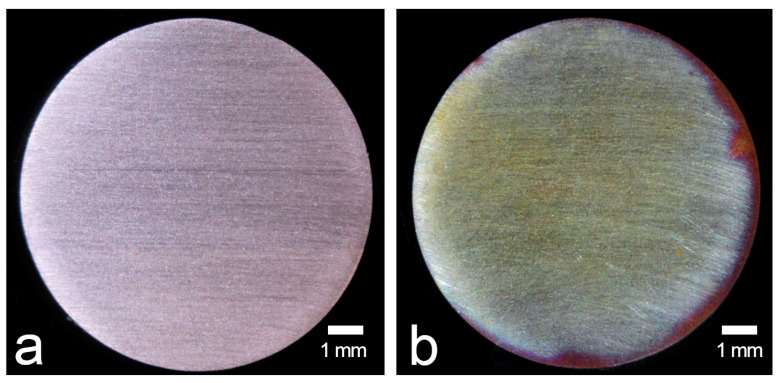
Copper specimens: (**a**) UH, unheated copper; (**b**) HT, heated copper. Digital photographic system: original magnification ×8.

A total of 44 specimens (a thickness of 3 mm) were cut from rod-like copper (a diameter of 10 mm). All specimens were wet-ground with silicon carbide abrasive paper (1500-grit, WetorDry Sheet, 3M Corp., St. Paul, MN, USA), subsequently cleaned with acetone, and air-dried (UH: unheated, Figure 1a). Half of the specimens (*n* = 22) were heated at 400 °C for 4 min in an electric furnace (Auto furnace QM-I, GC Corp., Tokyo, Japan) and were then allowed to cool to room temperature (HT: heated, Figure 1b). The processing conditions of HT were based on previous reports [8,9].

### 2.3. Preparation for Shear Bond Strength Testing

The specimens were further divided into two groups. Each group (*n* = 11) was either primed by MTU-6 or unprimed. MMA-TBB resin was filled into the stainless-steel ring (SUS303) placed around the bond area (a diameter of 5 mm). The polymerized specimens were immersed in distilled water at 37 °C for 24 h. The specimens were mounted in a shear bond testing jig and a steel mold was used to determine the shear bond strength with a mechanical testing device (Type 5567, Instron, Canton, MA, USA). The crosshead speed for the measurement was 0.5 mm/min. After testing, the failure modes of the bonded areas were classified with an optical microscope (8×, SZX9, Olympus Corp., Tokyo, Japan). Failure modes were classified into one of the following categories: CA, a combination of adhesive and cohesive failures; A, adhesive failure at the acrylic resin–metal interface.

### 2.4. Statistical Analysis

The results of shear bond strength testing were analyzed using descriptive statistics. A normal distribution was confirmed by the D’Agostino and Pearson omnibus test (GraphPad Prism 8, GraphPad software Inc., La Jolla, CA, USA). Further, Steel–Dwass test (Kyplot 5.0; KyensLab, Tokyo, Japan) based on the non-parametric Kruskal–Wallis test (Kyplot 5.0) were used to analyze the results. Statistical significance was set at *α* = 0.05.

### 2.5. X-Ray Photoelectron Spectroscopy (XPS) Analysis

Two types of specimen surfaces (HT and UH) were analyzed chemically using X-ray photoelectron spectroscopy (XPS; ESCA-3400, Shimadzu Corp., Kyoto, Japan) with Mg-Kα radiation. Wide- and narrow-scan spectra (Cu 2*p*, Cu *LMM*, O 1*s*) were acquired. Auger spectra, e.g., Cu *LMM*, are shown in different positions according to the type of radiation (AL-Kα or Mg-Kα).

## 3. Results

### 3.1. Shear Bond Strength Testing

The results of the D’Agostino and Pearson omnibus tests showed a normal distribution for all conditions; however, the results of the Bartlett test did not show homoscedasticity. The results of the Kruskal–Wallis test showed a significant difference (*P* < 0.001), and Steel–Dwass test showed that there was a significant difference among all groups (*P* < 0.05).

The median shear bond strength ranged from 2.3 to 28.3 MPa. These results are shown in Table 2. When primed with MTU-6, unheated copper (28.3 MPa) showed greater bond strength than heated copper (19.1 MPa). Shear bond strengths were markedly reduced when copper was unprimed. When unprimed, heated copper (4.1 MPa) showed greater bond strength than unheated copper (2.3 MPa).

**Figure 2:**
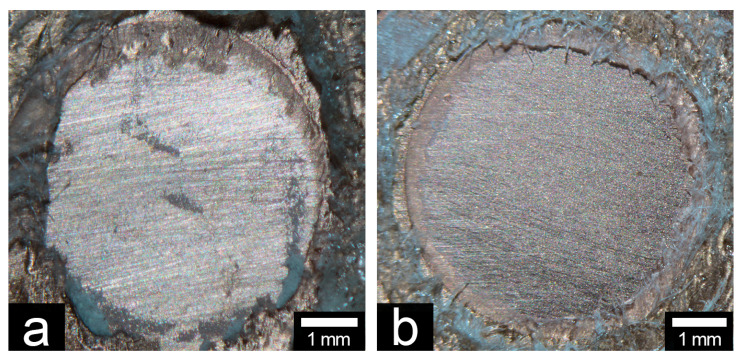
The representative debonded surfaces of coppers: (**a**) combination of adhesive and cohesive failures (CA, UH–MTU-6 group); (**b**) adhesive failure at the acrylic resin–metal interface (A, UH–unprimed group). Digital photographic system: original magnification ×8.

The results of the debonded surfaces observation are summarized in Table 2. Figure 2 shows the characteristically debonded copper surfaces of CA and A. Only one group (UH–MTU-6) showed a combination of adhesive and cohesive failures in all specimens. The rates of interfacial adhesion fracture in specimens increased as the shear bond strength decreased.

### 3.2. XPS Analysis

**Figure 3:**
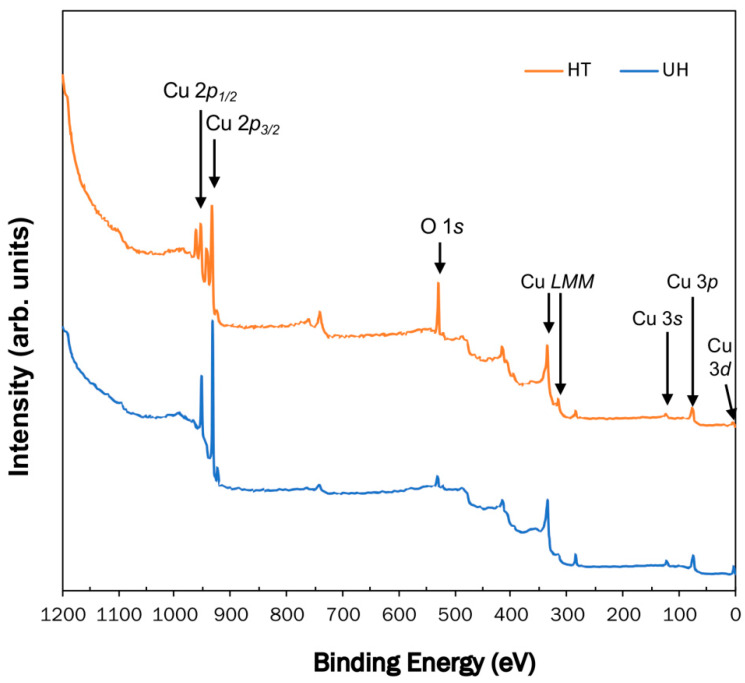
X-ray photoelectron spectroscopy (XPS) wide-scan spectra of the unheated Cu plate (UH) and heated Cu plate (HT).

The XPS results indicated that the copper surfaces were changed by heating. Figure 3 shows the wide scan spectrum of the copper surfaces of UH and HT. The copper surface under the HT condition showed that the peak of O 1*s* was stronger than the UH condition.

**Figure 4:**
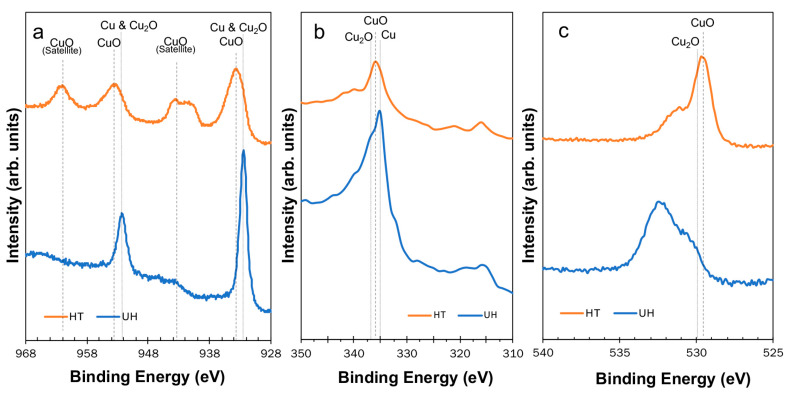
XPS narrow scan spectra of Cu *2p* region (**a**), Cu *LMM* (**b**), and O *1s* region (**c**) of the Cu plates.

Figure 4 shows narrow scan spectra for distinguishing the characteristics of copper surfaces. The peaks are listed in Table 3. The narrow scan spectra of the Cu 2*p* region are shown in Figure 4a. The unheated copper surface shows that peaks correspond to Cu_2_O (Cu^1+^) or Cu (Cu^0^), which are significantly narrower peaks at 932.4 eV and 952.2 eV. In contrast, the heated copper surface showed relatively broad peaks arising from CuO at 933.7 eV and 953.3 eV. In addition, characteristic satellite peaks arising from CuO were observed at 943.4 eV and 962.0 eV.

Figure 4b shows the narrow scan spectra of Cu *LMM* when Mg-K*α* radiation was used. The peak at 335 eV, seen on the unheated copper surface, is due to the band characteristic of Cu at Cu *LMM*. Similarly, the peak at 336 eV is due to the band characteristic of CuO, whilst the band characteristic of Cu_2_O is at 337 eV (Table 3).

Figure 4c shows the narrow scan spectra of O 1*s*. The peak at 529.7 eV seen on the heated copper surface is due to the band characteristic of the oxide in CuO. The peak of the band characteristic of the oxide contained in Cu_2_O (at about 530 eV) was included in the broad peaks, and was unable to be differentiated. 

## 4. Discussion

The results of shear bond strength testing showed that the combination of MTU-6 and unheated copper was the highest under the four conditions (Table 2). Groups primed with MTU-6 had greater bond strength than the unprimed groups. The effectiveness of MTU-6 against copper is similar to that reported in previous studies [5]. Notably, these results demonstrate that the bonding strength was significantly reduced by heating. When unprimed with MTU-6, heated copper had greater bond strength than unheated. Thus, the null hypothesis was rejected. Previous studies have shown that acidic functional monomers effectively adhere to the noble metal alloy containing small amounts of copper in oxidized form [8,9]. Even though these studies on the oxidation of noble metal alloys have been conducted prior to organic sulfur compounds being widely applied in dentistry, the effect of such sulfur-based compounds on the oxidation of copper remains unknown.

The surface properties were revealed by XPS. The XPS results of Cu_2_O and CuO were consistent with the standard spectra and experimental findings reported elsewhere [12,13,14,15,16,17,18]. The unheated copper surface revealed the states of Cu_2_O (Cu^1+^) or Cu via the narrow scan spectra of Cu 2*p* (Figure 4a and Table 3). The narrow scan spectra of Cu *LMM* and O 1*s* suggest that it is not Cu_2_O but instead Cu (Figure 4b,c and Table 3). However, the broad peaks of Cu *LMM* and O 1*s* do not necessarily deny that Cu_2_O does not exist on the unheated copper surface [19]. Unheated copper was examined with XPS immediately after the polishing process to analyze the copper surface under the same conditions used in the shear bond strength testing. In the report by Yamashita et al., Cu_2_O was detected from the debonded specimens of copper after thermocycling (20,000 cycles) using X-ray diffraction (XRD) [5]. It is therefore inferred that Cu changes to Cu_2_O when left following polishing. The surface of the heated copper was neither Cu nor Cu_2_O, but CuO as an oxide film which covered both Cu and Cu_2_O [1,2,3]. Tanaka et al. reported that a thick oxide film has an influence on adhesion using carboxylic anhydrides [8]. The surface of the copper showed CuO when heated, and significantly reduced the effect of MTU-6, demonstrating that oxidized copper reduced the effect of organic sulfur compounds.

Organic sulfur compounds adsorbed on the surface of noble metals by tautomerizing the structure contribute to chemical bonding between the acrylic resin and noble metal alloy [4,11]. In relation to this, not only Cu_2_O, but also CuO performed poorly. Copper is included in various dental noble metal alloys. A previous study suggested that the main component of the alloy and the structure of the functional group of the monomer to be polymerized strongly influence the compatibility of the alloy-functional monomer [5]. In recent years, it has been shown that airborne-particle abrasion with alumina may oxidize copper contained in noble metal alloys [12,18]. It was suggested that it performed better when using primers containing both an acidic functional monomer and an organic sulfur compound. Oxidized copper was revealed to reduce the effectiveness of organic sulfur compounds in this study. The result suggests that the oxidation of copper, including in noble dental alloys, also needs to be considered in the adhesion process.

## 5. Conclusions

The following conclusions can be drawn from this study: the copper surface showed that Cu (Cu^0^) or Cu_2_O (Cu^1+^) changed to CuO (Cu^2+^) upon heating, and this type of transformation significantly reduced the effect of MTU-6 in enhancing the adhesion of an acrylic resin onto such a metallic substrate. These results confirmed that it is necessary to take care of the copper oxide contained in noble metal alloys when using organic sulfur compounds.

## Figures and Tables

**Table 1:** Materials assessed.

Material/Trade Name	Manufacturer	Lot	Composition
Element metal			
Copper metal	Nilaco Corp., Tokyo, Japan	44225602	Cu 99.9 mass %
Primer			
Metaltite	Tokuyama Dental Corp., Tokyo, Japan	0382	MTU-6, ethanol
Luting material			
Super-Bond C&B Catalyst V	Sun Medical Co., Ltd., Moriyama, Japan	RG23F	TBB, TBB-O, hydrocarbon
Super-Bond C&B Opaque Ivory Powder	Sun Medical Co., Ltd., Moriyama, Japan	RM1	PolyMMA, titanium oxide
Methyl methacrylate	Tokyo Chemical Industry Co., Ltd., Tokyo, Japan	ZJ3WJIJ	MMA, 99.8%

MTU-6, 6-methacryloyloxyhexyl 2-thiouracil-5-carboxylate; MMA, methyl methacrylate; TBB, tri-*n*-butylborane; TBB-O, partially oxidized tri-*n*-butylborane.

**Table 2:** Shear bond strength (MPa) and failure modes after testing.

Treatment–Primer	Median	IQR	CA	A
UH–MTU-6	28.3 ^a^	0.8	11	0
HT–MTU-6	19.1 ^b^	8.2	6	5
UH–unprimed	2.3 ^c^	0.3	0	11
HT–unprimed	4.1 ^d^	0.9	1	10

*n* = 11; Superscript letters (a, b, c, and d) are used to indicate statistical results: same letters indicate results were not significantly different in shear bond strength among the groups (Steel–Dwass test; *P* > 0.05); IQR, interquartile range; CA, combination of adhesive and cohesive failures; A, adhesive failure at the acrylic resin–metal interface.

**Table 3:** Peaks of bonding energies (eV) and auger transition kinetic energy (eV).

Element	Peak Energy (eV)	Peak Assignment (Compound)	References
Cu 2*p3/2*	932.4	Cu_2_O or Cu	[13,14]
Cu 2*p1/2*	952.2	Cu_2_O or Cu	[13,14]
Cu 2*p3/2*	933.7	CuO	[13,14]
Cu 2*p3/2*	943.4	satellite peaks of CuO	[13,14]
Cu 2*p1/2*	953.3	CuO	[13,14]
Cu 2*p1/2*	962.0	satellite peaks of CuO	[13,14]
Cu *LMM*	335	Cu	[15,16]
Cu *LMM*	336	CuO	[15,16]
Cu *LMM*	337	Cu_2_O	[15,16]
O 1*s*	529.7	CuO	[13,14]
O 1*s*	530	Cu_2_O	[13,14]
